# Wood allocation trade‐offs between fiber wall, fiber lumen, and axial parenchyma drive drought resistance in neotropical trees

**DOI:** 10.1111/pce.13687

**Published:** 2020-02-03

**Authors:** Thomas A. J. Janssen, Teemu Hölttä, Katrin Fleischer, Kim Naudts, Han Dolman

**Affiliations:** ^1^ Department of Earth Sciences, Cluster Earth and Climate Vrije Universiteit Amsterdam Amsterdam The Netherlands; ^2^ Institute for Atmospheric and Earth System Research/Forest Sciences, Faculty of Agriculture and Forestry University of Helsinki Helsinki Finland; ^3^ Land Surface‐Atmosphere Interactions Technical University of Munich Freising Germany

**Keywords:** drought resistance, embolism, fibers, hydraulic conductivity, neotropical trees, parenchyma, wood density, xylem volume allocation

## Abstract

Functional relationships between wood density and measures of xylem hydraulic safety and efficiency are ambiguous, especially in wet tropical forests. In this meta‐analysis, we move beyond wood density per se and identify relationships between xylem allocated to fibers, parenchyma, and vessels and measures of hydraulic safety and efficiency. We analyzed published data of xylem traits, hydraulic properties and measures of drought resistance from neotropical tree species retrieved from 346 sources. We found that xylem volume allocation to fiber walls increases embolism resistance, but at the expense of specific conductivity and sapwood capacitance. Xylem volume investment in fiber lumen increases capacitance, while investment in axial parenchyma is associated with higher specific conductivity. Dominant tree taxa from wet forests prioritize xylem allocation to axial parenchyma at the expense of fiber walls, resulting in a low embolism resistance for a given wood density and a high vulnerability to drought‐induced mortality. We conclude that strong trade‐offs between xylem allocation to fiber walls, fiber lumen, and axial parenchyma drive drought resistance in neotropical trees. Moreover, the benefits of xylem allocation to axial parenchyma in wet tropical trees might not outweigh the consequential low embolism resistance under more frequent and severe droughts in a changing climate.

## Abstract 2

The hydraulic safety‐efficiency trade‐off in neotropical trees is strongly related to trade‐offs in wood volume allocation. Fiber wall volume benefits embolism resistance, fiber lumen contributes to sapwood capacitance and axial parenchyma is associated with higher hydraulic conductivity.

## 1 INTRODUCTION

Neotropical forests store large amounts of carbon in living biomass and are among the most species‐rich ecosystems on Earth (Gloor et al., 2012; Hoorn et al., 2010). General circulation models project a warming trend over South and Central America of 2–5°C by the end of the 21st century under the IPCC high‐end emission scenario (Lyra et al., 2017; Marengo et al., 2010). In addition, dry season precipitation is expected to decline (Boisier, Ciais, Ducharne, & Guimberteau, 2015; Malhi et al., 2009; Marengo et al., 2010), a trend that is ongoing since 1979 (Fu et al., 2013). Furthermore, there are signs that extreme episodic droughts are intensifying because of stronger Atlantic north‐south sea surface temperature gradients (Cox et al., 2008; Erfanian, Wang, & Fomenko, 2017). Episodic droughts, most notably in 2005, 2010, and 2015 have resulted in reduced tree growth and increased mortality in neotropical forests, specifically in the ever wet forest of the Amazon Basin (Feldpausch et al., 2016; Phillips et al., 2009; Rifai et al., 2018). Furthermore, increased drought and heat have been identified as possible drivers of a long‐term trend of increasing tree mortality in the Amazon Basin resulting in a reduction of the Amazon forest carbon sink strength (Brienen et al., 2015; McDowell et al., 2018). Despite the critical role of neotropical forests in driving future climate scenarios and their importance for biodiversity and human livelihoods, there are still large uncertainties surrounding the sensitivity of these forests to drought.

The tremendous biological and functional diversity found in tropical forest plant communities challenges the development of a robust theoretical framework that includes both the drivers of drought resistance and the conditions under which drought‐induced vegetation responses occur. For example, the vulnerability to drought‐induced mortality varies significantly among different species (Esquivel‐Muelbert et al., 2017), functional groups (Aleixo et al., 2019) and life forms (Nepstad, Tohver, David, Moutinho, & Cardinot, 2007). Interspecific differences in drought resistance can be attributed to properties of the trees water transporting tissue, i.e. the xylem sapwood. An evolutionary trade‐off between xylem water transport efficiency and xylem hydraulic and mechanical safety has been proposed to explain interspecific differences in drought resistance (Baas, Ewers, Davis, & Wheeler, 2004; Chave et al., 2009). However, recent studies show that generally, there is a weak trade‐off between hydraulic safety and efficiency, with many species showing both a low hydraulic safety and low hydraulic efficiency (Gleason et al., 2016; van der Sande, Poorter, Schnitzer, Engelbrecht, & Markesteijn, 2019). These findings limit the generalization and broader implementation of the safety‐efficiency trade‐off hypothesis in modeling frameworks and illustrate that the underlying mechanisms driving interspecific differences in drought resistance are still poorly understood in highly diverse tropical forests.

### 1.1 How do xylem hydraulic properties emerge from xylem traits?

Hydraulic efficiency often refers to the sapwood area specific hydraulic conductivity (*K*_*s*_), which is the rate of water transport through a given area of sapwood across a pressure gradient (Gleason et al., 2016; Tyree & Zimmermann, 2002). Another measure of hydraulic efficiency is sapwood capacitance, which is the amount of stored water that is released from the sapwood under a given pressure. Capacitance contributes to 5% to 30% of daily water use and enables high transpiration rates and stomatal conductance in the morning (Meinzer, James, Goldstein, & Woodruff, 2003; Oliva Carrasco et al., 2015). Furthermore, capacitance buffers the drop of leaf and branch xylem water potential during moments of high evaporative demand. In this way, capacitance helps to avoid plant desiccation that would initiate xylem embolism, leaf turgor loss and eventually drought‐induced mortality (Pratt & Jacobsen, 2017; Tyree, Engelbrecht, Vargas, & Kursar, 2003; Wolfe, 2017).

**Figure 1:**
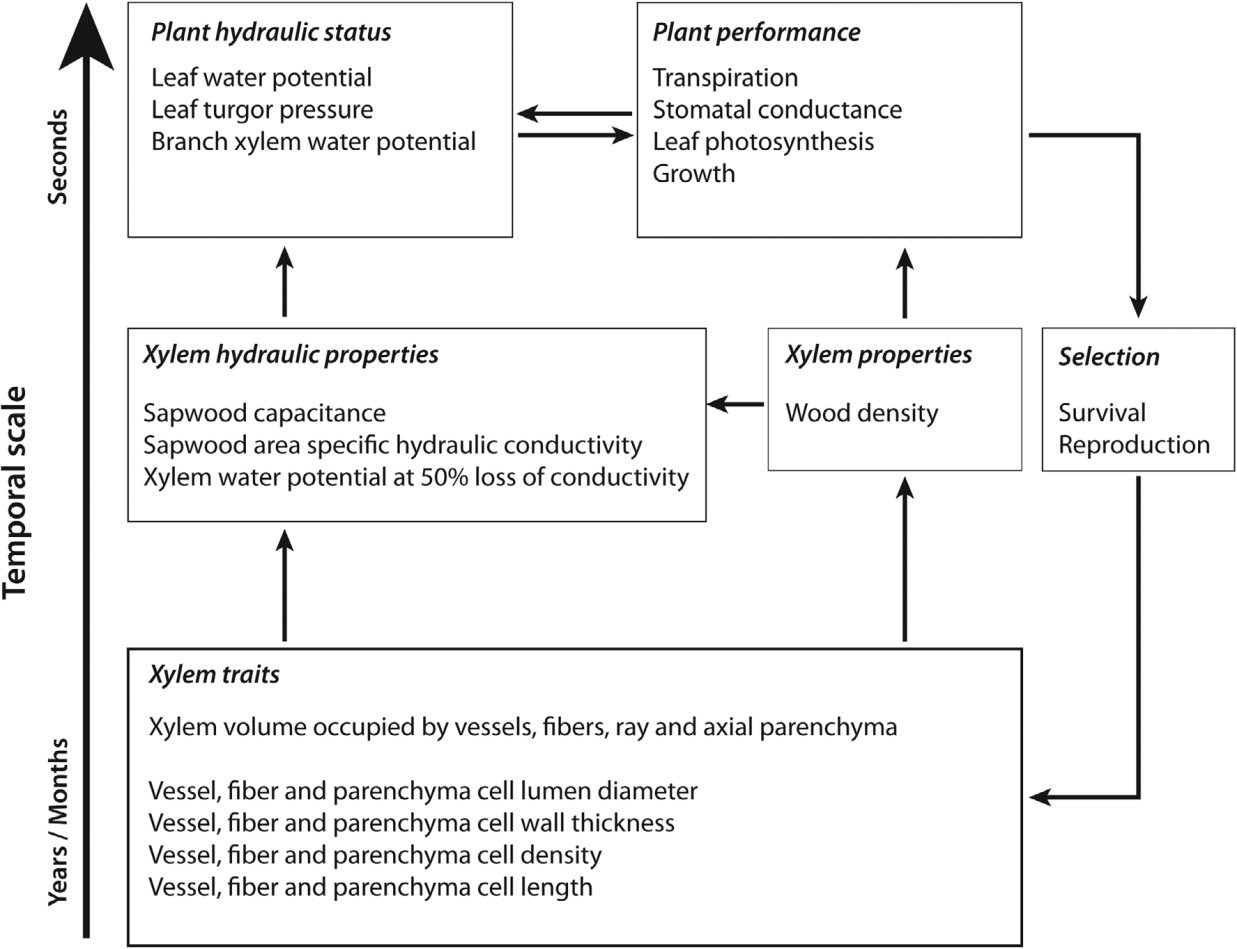
Hierarchical framework depicting how xylem traits drive xylem properties and xylem hydraulic properties that in turn determine plant hydraulic status and performance. Xylem traits generally operate on the individual cell and tissue level and on the developmental time scales of years and months. Plant performance and hydraulic status can act on relatively short time‐scales of minutes and seconds (e.g. opening and closure of the stomata)

Xylem hydraulic properties arise from a combination of xylem traits that are closely related to the anatomy of the xylem (Figure 1). This paper uses a hierarchical framework to assess the relationships between different measures that drive plant drought resistance (Lachenbruch & Mcculloh, 2014). Vessel diameter, length, and inter vessel pit properties are assumed to be key determinants of hydraulic conductivity and embolism resistance (Hacke & Sperry, 2001; Tyree & Sperry, 1989). According to the Hagen‐Poiseuille principle, the maximum hydraulic conductivity of a xylem vessel is proportional to the fourth power of the diameter of that vessel (Tyree & Zimmermann, 2002). Having slightly wider xylem vessels thus results in a considerable increase in hydraulic conductivity and water uptake efficiency (Gartner, Bullock, Mooney, Brown, & Whitbeck, 1990; Mcculloh et al., 2012; Méndez‐Alonzo, Paz, Zuluaga, Rosell, & Olson, 2012). However, wider vessels have also been related to thinner and more porous pit membranes between vessels which increase the risk of xylem embolism (Christman, Sperry, & Adler, 2009; Hacke, Sperry, Pockman, Davis, & McCulloh, 2001; Hacke, Sperry, Wheeler, & Castro, 2006; Mcculloh et al., 2012; Tyree & Sperry, 1989).

A common measure of xylem hydraulic safety is embolism resistance, often expressed as the xylem water potential at which 50% of the hydraulic conductivity is lost through embolism (*P*_50_). The difference between the minimum daily xylem water potential and *P*_50_ is the hydraulic safety margin, which is indicative of the amount of hydraulic risk a plant takes by approaching a water potential that initiates a dangerous increase of embolism (Meinzer, Johnson, Lachenbruch, McCulloh, & Woodruff, 2009). Reduced hydraulic conductance from embolism can in turn result in a drop of xylem water potential and more vessels becoming embolized (runaway embolism), eventually leading to tree mortality from hydraulic failure (Choat et al., 2018; Rowland et al., 2015; Sperry, Alder, & Eastlack, 1993; Tyree et al., 2003). It is important to stress that natural selection always acts on plant performance and not on individual traits or properties (Lachenbruch & Mcculloh, 2014). Therefore, we cannot conclude from a single hydraulic property whether or not a species or individual can be regarded as drought vulnerable or resistant as drought resistance emerges from the interaction of multiple hydraulic properties (Figure 1). It is still highly uncertain how hydraulic properties are driving drought resistance and how these properties relate to xylem traits.

### 1.2 Is there a relationship between xylem properties and plant life‐history?

Xylem properties reflect both a plant's life‐history strategy as well as its underlying hydraulic strategies (Baas et al., 2004; Chave et al., 2009). Wood density is regarded a useful proxy of performance measures related to life‐history such as stem diameter growth and tree longevity (Chave et al., 2009; Coelho de Souza et al., 2016) because of its relationships with biomechanical properties such as wood strength and stiffness (Putz, Phyllis Coley, Montalvo, & Aiello, 1983; Van Gelder, Poorter, & Sterck, 2006). Furthermore, wood density has also been associated with hydraulic properties such as hydraulic conductivity (Mcculloh et al., 2012; Meinzer, Campanello, et al., 2008), capacitance (Borchert, 1994; Meinzer et al., 2003; Meinzer, Campanello, et al., 2008) and resistance to embolism (Hacke et al., 2001; Markesteijn, Poorter, Paz, Sack, & Bongers, 2011; Sperry & Hacke, 2004).

Low density wood of tropical light‐demanding pioneer trees has been related to higher hydraulic conductivity and higher capacitance but also less resistance against embolism (Mcculloh et al., 2012; Meinzer, Campanello, et al., 2008; Meinzer, Woodruff, et al., 2008; Poorter et al., 2010; Sterck, Markesteijn, Toledo, Schieving, & Poorter, 2014). Relationships between wood density, plant performance, and hydraulic properties are used to benchmark plant functional types in ecosystem models, where wood density is used as a proxy of potential tree growth, xylem hydraulic conductivity, capacitance, and embolism resistance (Christoffersen et al., 2016; Powell et al., 2018; Sakschewski et al., 2016). However, these relationships remain ambiguous and conflicting results are often found, especially in wet tropical forests (De Guzman, Santiago, Schnitzer, & Álvarez‐Cansino, 2017; Powell et al., 2017; Santiago et al., 2018; Trueba et al., 2017). Therefore, the xylem traits that are driving the observed variations in wood density, xylem hydraulic safety and efficiency are still largely unresolved for many neotropical tree species.

### 1.3 How can xylem volume allocation affect xylem hydraulic properties and xylem traits?

In addition to vessels, the secondary xylem of angiosperms is made up of ray and axial parenchyma, fibers and sometimes tracheids. The principle function of ray parenchyma is the radial translocation and storage of non‐structural carbohydrates (NSCs), water, and nutrients. The axial parenchyma has been related to the storage of NSCs and water, contributing to sapwood capacitance (Borchert & Pockman, 2005; Plavcová & Jansen, 2015). Furthermore, the xylem axial parenchyma fraction also increases with increasing vessel diameter, suggesting a possible role of axial parenchyma in axial water transport (Morris et al., 2018; Zheng & Martínez‐Cabrera, 2013). Fiber walls contribute to mechanical strength and support xylem vessels to withstand implosion under high tension while fiber lumen contribute to water storage and sapwood capacitance (Hillabrand, Hacke, & Lieffers, 2019; Jacobsen, Ewers, Pratt, Paddock, & Davis, 2005; Pratt & Jacobsen, 2017; Pratt, Jacobsen, Ewers, & Davis, 2007). Some angiosperms species also have tracheids, or transitional cell forms between tracheids and vessels (Carlquist & Schneider, 2002). Tracheids make up most of the xylem in gymnosperms and combine a water transporting function with mechanical strength because of their relatively thick lignified cell walls (Sperry, Sano, Sikkema, Feild, & Hacke, 2007).

Like other xylem properties, wood density emerges from the interaction of different xylem traits and the partitioning of wood volume to the different tissues. While hydraulic efficiency and safety are often attributed to vessel traits (Hoeber, Leuschner, Köhler, Arias‐Aguilar, & Schuldt, 2014; Markesteijn et al., 2011; Méndez‐Alonzo et al., 2012), wood density in tropical trees is found to be mainly driven by the partitioning of wood volume to axial parenchyma, fiber walls, and fiber lumen (McDonald, Williamson, & Wiemann, 1995; Ziemińska, Butler, Gleason, Wright, & Westoby, 2013). Therefore, the use of wood density as a proxy of xylem hydraulic safety and efficiency has a weak functional basis (Lachenbruch & Mcculloh, 2014; Patiño et al., 2012). Trade‐offs in xylem volume allocation to different tissues are underlying fundamental trade‐offs between biomechanics, water transport efficiency and storage of water, nutrients, and carbohydrates (Pratt & Jacobsen, 2017).

While the importance of vessel traits in driving hydraulic properties and plant performance in neotropical trees is widely recognized and studied (e.g. Christensen‐Dalsgaard, Ennos, & Fournier, 2007; Hoeber et al., 2014; Markesteijn, Poorter, Bongers, et al., 2011; Méndez‐Alonzo et al., 2012) the importance of fibers and parenchyma in this respect has received less attention (Fortunel, Ruelle, Beauchêne, Fine, & Baraloto, 2014; Osazuwa‐Peters, Wright, & Zanne, 2017; Poorter et al., 2010). This study fills this gap, motivated by the evidence from Mediterranean climate shrub ecosystems that trade‐offs in xylem volume allocation are driving interspecific variability in embolism resistance and sapwood capacitance (Jacobsen et al., 2005; Jacobsen, Esler, Brandon Pratt, & Ewers, 2009; Pratt et al., 2007). Here, we will test the hypothesis that in neotropical tree species, xylem volume allocation to fiber and vessel walls is driving embolism resistance while wood volume allocation to fiber lumen, ray and axial parenchyma contributes to sapwood capacitance and specific conductivity. Furthermore, we expect that wood density increases with fiber and vessel wall fractions and declines with fiber lumen and axial parenchyma fractions. Finally, we hypothesize that xylem volume allocation trade‐offs are underlying the evolutionary trade‐off between xylem hydraulic safety and efficiency. We aim to move beyond wood density as a driver of hydraulic properties and as such improve our understanding of xylem volume allocation in determining drought resistance.

## 2 MATERIALS AND METHODS

The data compilation focused on observations of xylem properties and traits related to hydraulic functioning and drought resistance of angiosperm trees from the lowland humid forest of Amazonia sensu lato (the Amazon Basin and the Guianas) and Central America, which are similar in climate and phylogeny (Eva et al., 2005; Slik et al., 2018). In addition, observations were retrieved from the Cerrado and tropical dry forests of South and Central America stretching from 25° North to 25° South. The observations were stored in a database that also included the species and genus name, site location and the characteristics of the individual: height, diameter at breast height (DBH), etc. (Database S1). Species growth form was largely derived from *the plant growth form dataset for the New World* (Engemann et al., 2016). The taxonomic status of the original genus and species names were assessed using *The Plant List*—*version 1.1* (http://www.theplantlist.org) in the *R* package *Taxonstand* (Cayuela, Granzow‐de la Cerda, Albuquerque, & Golicher, 2012) and the original genus and species names were replaced by the accepted names when necessary.

Individual observations were complimented by species level observations from existing databases such as the global wood density database (Zanne et al., 2009), the global parenchyma database (Morris et al., 2016) and the RAINFOR Amazon‐wide tree biomass and tree growth databases (Coelho de Souza et al., 2016; Fauset et al., 2015). Also the wood density databases of the Centre de coopération internationale en recherche agronomique pour le développement (CIRAD) and of The Food and Agriculture Organization of the United Nations (FAO) were consulted (Vieilledent et al., 2018). The core dataset counts 11956 entries of individual observations on 3909 species from 902 genera and 155 plant families that were retrieved from 346 published studies conducted at 110 sites in Central and South America.

Vines and lianas were omitted from the analysis as well as species from the *Cactaceae* and *Winteraceae* families and the *Clusia* genus. The reason for omitting these species from the meta‐analysis was that some species from the *Cactaceae* and *Winteraceae* are vesselless and could therefore not be compared with vessel bearing species (Feild & Holbrook, 2000; Hacke et al., 2007). Species from the *Clusia* genus were considered outliers because of very low specific hydraulic conductivity (Feild & Holbrook, 2000). These records were, however, maintained in the database. The number of individual observations and individual species and genera available in the database varied significantly between different measures (Table S1). Linear and non‐linear regression models were fitted using both genus and species averaged data. Genus averaged data was only used when there was no data available for a single species in that genus and was otherwise omitted.

Many studies did not report the maximum sapwood area specific hydraulic conductivity (*K*_*s*_) and native state (with naturally occurring embolism) sapwood area specific hydraulic conductivity consistently. For studies that only reported native state sapwood specific hydraulic conductivity, we estimated the native state loss of hydraulic conductivity from its sigmoidal relationship with *P*_50_ (Figure S1). If *P*_50_ was also not available, the average value of native state embolism of 31% of maximum *K*_*s*_ observed across studies was used to estimate *K*_*s*_. About half of the *K*_*s*_ data used in the analyses was estimated from native state hydraulic conductivity (Table S1). Furthermore, we estimated the minimum observed terminal branch xylem water potential for 329 tree species (Table S1) from a strong correlation between this measure and the minimum leaf water potential (Figure S2).

**Figure 2:**
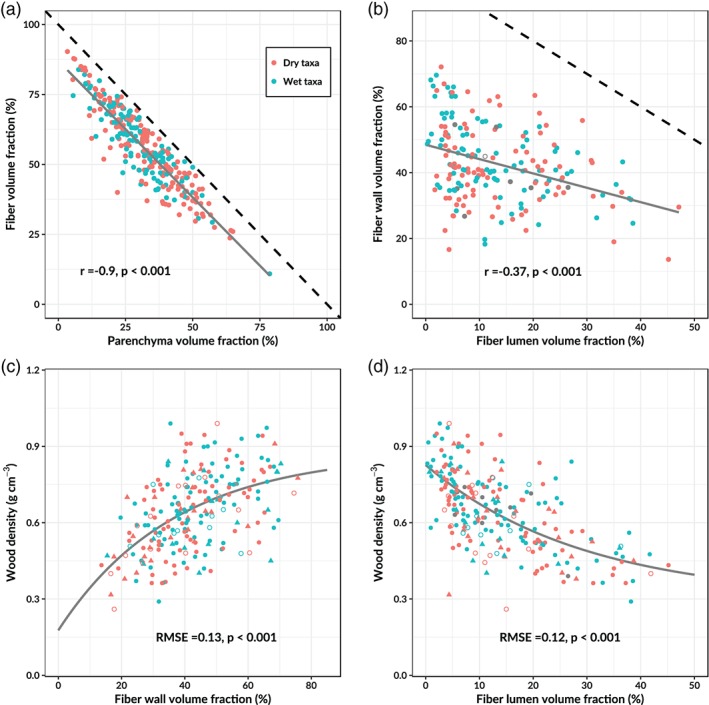
Trade‐offs in xylem volume allocation and their relationships with wood density. Different regression lines were fitted for species and genera affiliated to dry (red) and wet environments (blue) when the slope and/or intercept of the regression differed significantly (ANCOVA *p* < .05). Species averages are filled and genus averages are open circles. In (a) and (b), the black dashed lines indicate the “packing limit” or the maximum (100%) volume. Filled triangles indicate that for these species the fiber fraction was estimated from the parenchyma volume fraction

For 135 species the parenchyma but not the fiber volume fraction was available (Table S1). For these species we estimated the fiber volume fraction from the strong trade‐off relationship observed between fiber and parenchyma volume (Figure 2a). Subsequently, the fiber and vessel wall and lumen fractions were estimated for species where the mean lumen diameter and wall thickness were available. The fiber and vessel lumen fraction was estimated as:

$$A_{\mathrm{clf}}=\frac{\pi {\left(\frac{D_{\mathrm{cl}}}{2}\right)}^{2}}{\pi {\left(\frac{D_{c}}{2}\right)}^{2}}A_{\mathrm{cf}}$$

where *D*_cl_ is either the fiber or vessel cell lumen diameter (μm), *D*_c_ the total cell diameter (μm), and *A*_cf_ the fiber or vessel total volume fraction (%). The fiber or vessel wall fraction was then calculated as the difference between the total fiber or vessel volume fraction and the lumen fraction.

Drought resistance is defined here as the ability to survive and reproduce in an environment that experiences recurrent seasonal drought. We use the species specific water deficit affiliation (WDA) (Esquivel‐Muelbert et al., 2017, 2018) and drought‐induced mortality to quantify drought resistance. The WDA is the maximum climatological water deficit (mm) of an area in which a species has its center of gravity in terms of distribution. Drought induced mortality is the increase of the tree mortality rate following drought, relative to the baseline mortality without drought (da Costa et al., 2010; Nepstad et al., 2007). WDA and drought‐induced mortality have been shown to be related and are, despite lacking a physiological basis, expected to be good indicators of tree species drought resistance (Esquivel‐Muelbert, Baker, et al., 2017).

The species‐specific water deficit affiliation (WDA) values were retrieved from the datasets of Esquivel‐Muelbert, Galbraith, et al. (2017); Esquivel‐Muelbert et al. (2018). The WDA is the average cumulative water deficit (CWD) value of the area in which the specific species has its center of gravity in terms of distribution (see Esquivel‐Muelbert, Galbraith, et al., 2017 for a description of the methods). CWD is calculated following Aragão et al. (2007) and is either negative (water deficit) or zero:

$$\text{If}\;{\mathrm{CWD}}_{t-1}-E_{t}+P_{t}<0;$$

$$\text{then}\;{\mathrm{CWD}}_{t}={\mathrm{CWD}}_{t-1}-E_{t}+P_{t};$$

$$\text{else}\;{\mathrm{CWD}}_{t}=0$$

where CWD_*t*−1_ is the CWD in the previous month and *E*_*t*_ and *P*_*t*_ are the evapotranspiration and precipitation in the current month, respectively. To estimate the WDA for species where WDA was not available, the average WDA for each of the 110 sites in the database was calculated. The missing species‐specific WDA values were estimated using the average of the site‐specific WDA values in which the species occurred. In most analyses, species and genera were grouped into wet and dry taxa with the boundary chosen at a WDA of −200 mm, a CWD value at which seasonally dry forest and savanna are starting to co‐occur with wet evergreen forest in South and Central America (Malhi et al., 2009).

Drought mortality data was retrieved from different published drought experiments on seedlings and saplings in Panama (Engelbrecht et al., 2007; Kursar et al., 2009; Tyree et al., 2003) and from juvenile and adult trees in the throughfall exclusion experiments at Tapajós and Caxiuanã in Brazil (da Costa et al., 2010; Nepstad et al., 2007). In addition, we included drought mortality data from saplings and adult trees from a natural drought in Panama (Condit, Hubbell, & Foster, 1995). Drought‐induced mortality was then calculated following Esquivel‐Muelbert, Baker, et al. (2017) as the absolute difference between mortality during drought and baseline mortality (without drought). Species averaged baseline mortality in more natural settings and over longer periods was sometimes found to be higher than total mortality during drought, which was rarely the case in short term high intensity drought experiments. Around 24% of the observations showed a higher mortality in the control compared to the drought treatment from which 70% of these observations were retrieved from the natural drought event in Panama (Condit et al., 1995). To account for this, we set every tree species with a drought‐induced mortality index lower than zero to zero and then normalized the drought‐induced mortality values in every study from zero (low mortality) to one (high mortality).

## 3 RESULTS

### 3.1 Drivers of wood density and trade‐offs in wood allocation

A strong trade‐off emerged between parenchyma volume fraction and the fiber volume fraction (Figure 2a). This relationship is mainly driven by the increase of axial parenchyma with increasing parenchyma fraction (not shown, *r* = 0.86, *p* < .001) and less by an increase of ray parenchyma with increasing parenchyma fraction (not shown, *r* = 0.37, *p* < .001). The highest fractions of wood allocated to axial parenchyma (>40%) were observed in genera of the Malvaceae such as *Ceiba*, *Sterculia*, and *Ochroma* and the Fabaceae, including *Lonchocarpus*, *Ormosia*, and *Inga*. The strong correlation between fiber and parenchyma volume fractions also implies that the remaining xylem volume occupied by vessels was not as variable as the volume occupied by fibers and parenchyma.

We found another trade‐off between xylem volume allocated to fiber lumen and fiber walls (Figure 2b). The fiber lumen‐wall allocation trade‐off is the main driver of interspecific differences in wood density across the dataset (Figure 2c,d). High wood density genera such as *Anadenanthera*, *Guaiacum*, and *Myroxylon* allocate 70–75% of their wood volume to fiber walls and only 3–5% to fiber lumen. Conversely, low wood density genera such as *Poulsenia*, *Schizolobium*, and *Simarouba* allocate 20–30% of their wood volume to fiber walls and 35–45% to fiber lumen. From the remaining xylem volume fractions, only ray parenchyma fraction and vessel wall fraction showed marginally significant relationships with wood density. Wood density declined with ray parenchyma (not show, *r* = −0.15, *p* < .05) and increased with vessel wall volume fraction (not shown, *r* = 0.19, *p* < .05). These results suggest that there are strong packing space related trade‐offs between wood volume allocation to parenchyma and fibers and between fiber wall and lumen that are driving interspecific differences in wood density.

### 3.2 Xylem properties and traits related to hydraulic efficiency

**Figure 3:**
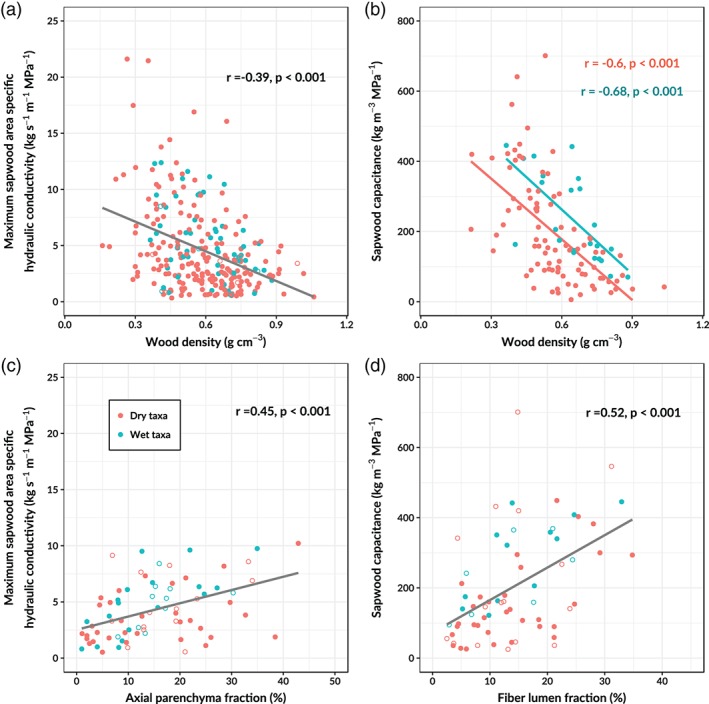
Relationships between measures of hydraulic efficiency and xylem properties and traits. Different regression lines were fitted for species and genera affiliated to dry (red) and wet environments (blue) when the slope and/or intercept of the regression differed significantly (ANCOVA *p* < .05). Species averages are filled and genus averages are open circles [Color figure can be viewed at https://wileyonlinelibrary.com]

Wood density was significantly related to the two measures of hydraulic efficiency (Figure 3). Sapwood area specific hydraulic conductivity (*K*_*s*_) declined significantly with increasing wood density, although many taxa showed a low wood density and a low *K*_*s*_ (Figure 3a). This suggests that wood density is a weak proxy of *K*_*s*_ in neotropical tree taxa. Sapwood capacitance also declined with increasing wood density in both wet and dry taxa (Figure 3b). However, wet tree species show on average a higher capacitance (~100 kg m^−3^ MPa^−1^) for a given wood density compared to dry species.

The variability in *K*_*s*_ among tree taxa could partly be explained by the xylem volume allocation to axial parenchyma, as *K*_*s*_ increased with increasing axial parenchyma fraction (Figure 3c). Furthermore, xylem traits related to hydraulic conductivity such as the length of the longest continuous vessel (Figure S3) and the mean vessel diameter (not shown, *r* = 0.26, *p* < .001) also increased with increasing axial parenchyma fraction. These results suggest that large xylem volume fractions of axial parenchyma are associated with the presence of wide and long xylem vessels.

Sapwood capacitance was found to be most dependent on the fiber lumen fraction (Figure 3c). As sapwood capacitance increased with increasing fiber lumen fraction, it declined with increasing fiber wall fraction (not shown, *r* = −0.33, *p* < .01). Furthermore, sapwood capacitance also declined with an increase of vessel wall fraction (not shown, *r* = −0.32, *p* < .05). There were no significant relationships between sapwood capacitance and other xylem volume fractions.

### 3.3 Xylem properties and traits related to hydraulic safety

**Figure 4:**
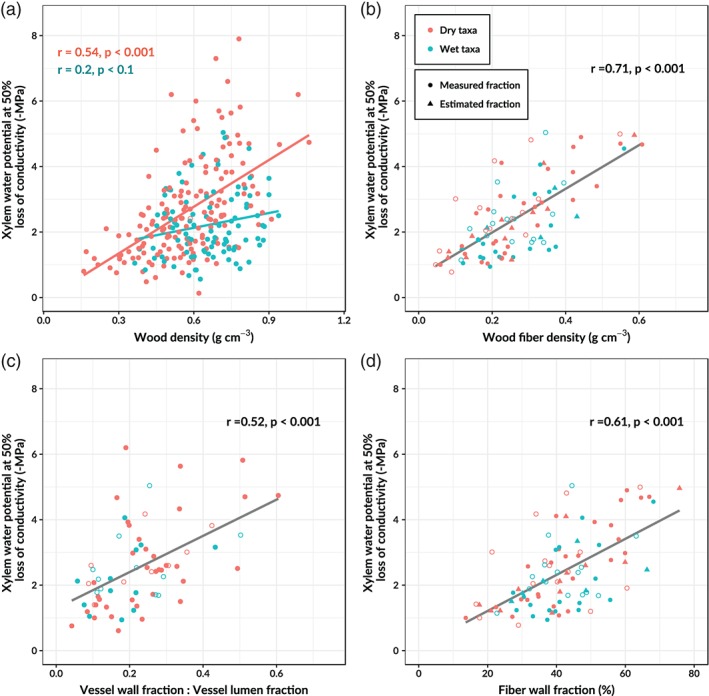
Relationships between xylem embolism resistance and xylem properties and traits. Different regression lines were fitted for species and genera affiliated to dry (red) and wet environments (blue) when the slope and/or intercept of the regression differed significantly (ANCOVA *p* < .05). Species averages are filled and genus averages are open circles. In panel (b), the fiber wall volume fraction was used to estimate the wood fiber density. Filled triangles indicate that for these species the fiber fraction was estimated from the parenchyma volume fraction [Color figure can be viewed at https://wileyonlinelibrary.com]

Relationships between wood density and xylem embolism resistance differed between taxa of wet and dry environments (Figure 4). We find that the *P*_50_ (MPa) increases with increasing wood density in both wet and dry taxa (Figure 4a). This suggests that xylem embolism resistance generally increases with increasing wood density. However, both the slope and the intercept of this relationship differed significantly between wet and dry taxa (ANCOVA, *p* < .001). The increase of embolism resistance with wood density is more than three times as steep in dry taxa (slope = 4.76) compared to wet taxa (slope = 1.51). Contrary to the relationship between *P*_50_ and overall wood density, the increase of *P*_50_ with wood fiber density (grams fibers per cm^3^ wood volume) is similar in species of both wet and dry environments (Figure 4b). Furthermore, we find that *P*_50_ does not increase with increasing wood parenchyma density (not shown) and even declines with increasing wood axial parenchyma density (Figure S4). This suggests that only wood mass invested in fiber walls contributes to hydraulic safety while wood mass invested in parenchyma does not, possibly explaining the weak relationship between wood density and *P*_50_ in wet tropical tree taxa.

We find that *P*_50_ increases with increasing vessel wall fraction to vessel lumen fraction ratio, which is a measure of the vessel bending resistance (Figure 4c). Furthermore, *P*_50_ increases with increasing fiber wall fraction (Figure 4d), with increasing sum of the fiber wall and vessel wall volume fractions (not shown, *r* = 0.67, *p* < .001) and vessel wall fraction alone (not shown, *r* = 0.30, *p* < .05) and declines with fiber lumen fraction and axial parenchyma fraction (not shown, *r* = −0.32, *p* < 0.01 and *r* = −0.37, *p* < .01, respectively). There was no significant relationship between *P*_50_ and ray parenchyma fraction or vessel lumen fraction. These results suggest that compared to embolism vulnerable taxa, embolism resistant taxa are associated with higher fiber and vessel wall fractions and lower fiber lumen and axial parenchyma fractions.

### 3.4 Wood density specific hydraulic safety and drought resistance

**Figure 5:**
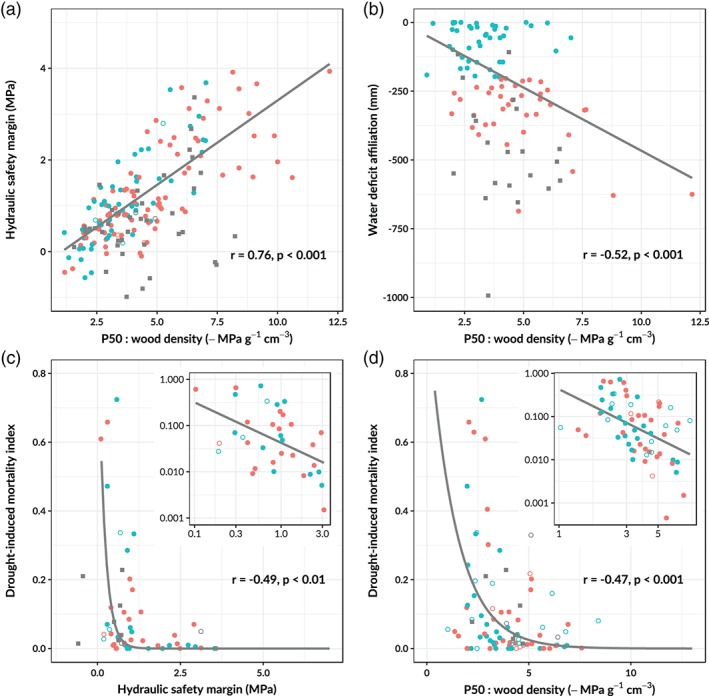
Relationships between measures of drought resistance and xylem hydraulic properties. Different regression lines were fitted for species and genera affiliated to dry (red) and wet environments (blue) when the slope and/or intercept of the regression differed significantly (ANCOVA *p* < .05). Species averages of evergreen tree species are filled and genus averages are open circles. Grey squares represent drought deciduous taxa that allow a more variable and narrow safety margin without risking dangerous increases in embolism. Deciduous taxa were for this reason omitted from the regression analysis. Insets in (c) and (d) are the same data on a log–log scale. Taxa with a drought mortality index of zero were omitted from the log‐log plots as they cannot be plotted on a logarithmic scale [Color figure can be viewed at https://wileyonlinelibrary.com]

The variability in *P*_50_ for a given wood density was found to be an important indicator of hydraulic behavior and drought resistance. We used the ratio of *P*_50_ (MPa) and wood density as a measure of wood density specific hydraulic safety (Figure 5). As the increase of wood density is associated with a decline in sapwood capacitance (Figure 3b) and therefore a decline in the buffering of branch xylem water potential, the increase of wood density has to be compensated by an increase in embolism resistance to maintain a safe margin between xylem water potential and *P*_50_ (the hydraulic safety margin). Indeed, we find that wood density specific hydraulic safety is strongly correlated to the hydraulic safety margin in evergreen tree taxa (Figure 5a). Deciduous trees keep a more variable and sometimes negative safety margin (Figure 5, grey squares) as they can easily shed leaves when leaves and branches desiccate, according to the so‐called hydraulic fuse hypothesis (Wolfe, Sperry, & Kursar, 2016).

Both the safety margin and the wood density specific hydraulic safety could partly explain the observed interspecific differences in drought resistance. The first measure of drought resistance, the water deficit affiliation (WDA) declined with increasing wood density specific hydraulic safety in evergreen tree taxa (Figure 5b). Furthermore, drought‐induced mortality increased with a declining safety margin and a decline of wood density specific hydraulic safety (Figure 5c,d). Finally, drought‐induced mortality was not significantly related to wood density alone but did decrease with *P*_50_ (*r* = −0.35, *p* < .01). This suggests that taxa with a high embolism resistance relative to their wood density are characterized by a wide safety margin, can survive in relatively dry environments and are highly resistant to drought‐induced mortality. Inversely, this implies that taxa confined to wet environments are characterized by a low wood density specific hydraulic safety and a narrow safety margin, which makes them unable to survive in seasonally dry environments.

### 3.5 Wood volume allocation, embolism vulnerability and plant life‐history

**Figure 6:**
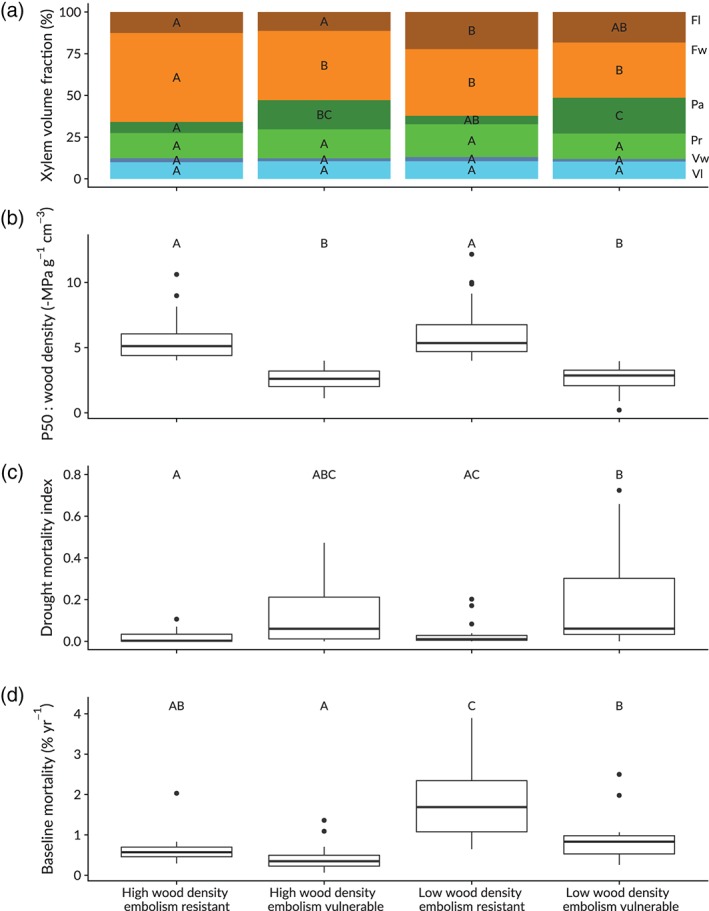
The effects of wood volume allocation to fiber lumen and walls (*F*_l_ & *F*_w_), axial and ray parenchyma (*P*_a_ & *P*_r_) and vessel walls and lumen (*V*_w_ & *V*_l_) on tree drought resistance and life‐history. Capital letters indicate a significant (Tuckey HSD *p* < .05) difference between the groups. Species averaged baseline mortality data was directly retrieved from the RAINFOR Amazon wide forest inventory database (Coelho de Souza et al., 2016) [Color figure can be viewed at https://wileyonlinelibrary.com]

To see how xylem volume allocation differs between embolism vulnerable and embolism resistant tree species, we separated the species into four categorical groups based on wood density and wood density specific hydraulic safety (Figure 6). Tree species were considered embolism resistant when the *P*_50_: wood density was 4.0 MPa g^−1^ cm^−3^ or higher and were otherwise considered embolism vulnerable. The value of 4.0 MPa g^−1^ cm^−3^ was chosen because drought‐induced mortality starts to increase exponentially when *P*_50_: wood density becomes lower than 4.0 MPa g^−1^ cm^−3^ (Figure 5d). Subsequently, the median wood density (~0.65 g cm^‐3^) was used to separate the groups again into a low and high wood density group. These groups differed significantly (Tukey HSD *p* < .05) in the xylem volume allocation to fiber lumen, fiber walls, and axial parenchyma (Figure 6a). We find that low wood density tree species invest a relatively large proportion of their xylem volume to fiber lumen, which in fact is the reason for their low wood density (Figure 2c,d). High wood density embolism resistant species invest a relatively large proportion of their xylem in fiber walls at the expense of fiber lumen and axial parenchyma. Embolism vulnerable tree species with high and low wood density invest a relatively large proportion of xylem volume to axial parenchyma (Figure 6a).

We find that this investment of wood volume to axial parenchyma is associated with a low wood density specific hydraulic safety (Figure 6b) resulting in a high drought‐induced mortality (Figure 6c). Interestingly, embolism sensitive tree species experience a relatively low baseline mortality (Figure 6d). Furthermore, tree transpiration, stomatal conductance, photosynthesis, and stem growth all decline with increasing wood fiber density (Figure S5). This suggests that xylem volume investment in axial parenchyma might be related to tree longevity as a preferential allocation of volume to axial parenchyma seems to be associated with a low baseline mortality (Figure 6d). Finally, we find that the estimated aboveground biomass and number of tree stems in the Amazon Basin from the RAINFOR plots (Fauset et al., 2015) are not evenly distributed over the four categorical groups (Figure S6). The summed above ground biomass from tree species in the high wood density embolism sensitive group represents more than half of the total aboveground biomass in the Amazon Basin.

## 4 DISCUSSION

### 4.1 Is wood density driving hydraulic efficiency?

Xylem volume allocation trade‐offs, mainly between fiber walls, fiber lumen, and axial parenchyma were found to drive interspecific variability in wood density and xylem properties related to hydraulic safety and efficiency. While wood density is often not directly related to the more fundamental hydraulic traits and properties, interacting xylem traits that influence both wood density and hydraulic properties can result in strong correlations between wood density, xylem embolism resistance (*P*_50_) and sapwood area specific hydraulic conductivity (*K*_*s*_) (Figure 1). Wood density is therefore often used as a proxy for these hydraulic traits and properties (Christoffersen et al., 2016; Lachenbruch & Mcculloh, 2014). In this meta‐analysis we moved beyond wood density as a driver of hydraulic properties and identified the xylem traits that are behind the observed relationships.

We find that fiber wall and lumen fractions show the strongest correlations with wood density of all the tissue fractions (Figure 2c,d). These results are in agreement with our hypothesis and confirm earlier findings from Australia and French Guiana that variations in fiber wall and lumen fractions drive observed variations in wood density across tropical trees (Fortunel et al., 2014; Ziemińska et al., 2013). Variations in axial parenchyma volume fractions have been found to explain radial changes in wood density within the trunk of individual trees (McDonald et al., 1995). However, contrary to our expectations, we found that axial parenchyma volume fractions could not explain interspecific differences in wood density. Furthermore, we confirm previous findings from neotropical rainforest (Christoffersen et al., 2016) and globally (Gleason et al., 2016), that wood density is a poor proxy of *K*_*s*_ across neotropical tree taxa (Figure 3a). However, we should note that considerable uncertainty is introduced in the estimation of maximum *K*_*s*_ in this meta‐analysis which could explain the scatter in the relationship between wood density and *K*_*s*_ (Figure 3a). Nonetheless, our results and previous work suggest that specific conductivity, which is attributed to vessel properties (Hoeber et al., 2014; Markesteijn, Poorter, Bongers, et al., 2011; Méndez‐Alonzo et al., 2012) is largely decoupled from the allocation of xylem volume to fiber walls and lumen that is driving interspecific differences in wood density.

The results from our meta‐analysis do suggest a positive association between *K*_*s*_ and axial parenchyma (Figure 3c). In addition, we confirm earlier findings that xylem volume allocated to axial parenchyma correlates positively with vessel diameter (Morris et al., 2018; Zheng & Martínez‐Cabrera, 2013). However, to our knowledge the positive relationship between measured sapwood‐area specific hydraulic conductivity and xylem axial parenchyma fraction has not been described previously. Finally, we found a strong positive relationship between the length of the longest continuous vessel, which is found to be positively related to *K*_*s*_ (Markesteijn, Poorter, Bongers, et al., 2011), and the xylem axial parenchyma fraction. Previous work on temperate angiosperms found that roughly half of the total resistivity in vessels originates from water that passes through the pit membrane pores that interconnect the vessels (Hacke et al., 2006). The passing of water between vessels can be expected to be considerably less in longer vessels, introducing less resistivity and thus a higher specific conductivity in xylem with long continuous vessels.

The mechanisms behind the positive relationship between vessel bordering (paratracheal) axial parenchyma and *K*_*s*_ are presently unclear. A role of paratracheal parenchyma cells in the refilling of embolized vessels has been suggested (Secchi, Pagliarani, & Zwieniecki, 2017; Tyree, Salleo, Nardini, Assunta Lo Gullo, & Mosca, 2002). Furthermore, paratracheal axial parenchyma cells are responsible for the loading of cations into the xylem vessels regulating the transpiration flow (de Boer & Wegner, 1997). Additionally, a recent study found that paratracheal parenchyma cells load insoluble surfactants (phospholipids) into the transpiration flow, possibly to reduce the expansion of emboli in the vessels and prevent cavitation (Schenk et al., 2017). Further experimental research is needed to identify the exact mechanisms that are driving the positive relationships between xylem volume allocated to axial parenchyma, vessel lumen diameter and length and sapwood area specific hydraulic conductivity.

This meta‐analysis shows for the first time that in neotropical trees, fiber lumen volume is the main contributor to sapwood capacitance (Figure 3d), as was previously found in other environments (Jupa, Plavcová, Gloser, & Jansen, 2016; Pratt et al., 2007). This mechanism explains the negative relationship between wood density and sapwood capacitance (Chave et al., 2009; Christoffersen et al., 2016; Meinzer, Campanello, et al., 2008; Pratt et al., 2007). Contrary to our hypothesis, we did not find a relationship between axial parenchyma volume fraction and sapwood capacitance. This suggests that across neotropical trees a high fraction of axial parenchyma is not always related to high capacitance as has been suggested previously (Borchert & Pockman, 2005). Sapwood capacitance is often estimated from the linear part of the moisture release curve. This represents the water that is released under low pressure, mainly from the vessel and fiber lumen and intercellular space (Jupa et al., 2016; Pratt & Jacobsen, 2017). Water released from the living parenchyma cells (elastic storage) does not contribute to this initial water release (Jupa et al., 2016). This mechanism may explain why sapwood capacitance and axial and ray parenchyma fractions were not correlated. The ecological importance of capacitance from parenchyma tissue in neotropical trees remains unresolved. The separation of capacitance into “fast” and “slow” capacitance from moisture release curves in tropical species could provide more insight into the role of parenchyma tissue in driving stem water storage (Jupa et al., 2016).

### 4.2 Is wood density driving hydraulic safety?

The relationship between wood density and *P*_50_ was weak, especially in tree taxa from wet tropical forests (Figure 4a). We find that the estimated wood fiber density, which is the product of the fiber wall volume fraction and wood density, shows a strong correlation with *P*_50_ across both wet and dry tropical tree taxa (Figure 4b). Although the wood fiber density is a simplification of the actual amount of wood mass allocated to fiber walls, the strong relationship between *P*_50_ and the estimated wood fiber density suggest a possible important role of wood mass invested in fiber walls in determining xylem hydraulic safety (Hacke et al., 2001; Hacke & Sperry, 2001; Jacobsen et al., 2005; Pratt et al., 2007). Contrastingly, wood mass allocated to axial parenchyma was negatively related to *P*_50_ (Figure S4). This finding could explain why evergreen tree species with high wood density from wet tropical forests are not necessarily drought resistant (Christoffersen et al., 2016; De Guzman et al., 2017; Poorter et al., 2010; Powell et al., 2017; Santiago et al., 2018).

The mechanical support of the vessels can either originate from a high vessel bending resistance (Figure 4c) or the presence of a strong fiber matrix supporting the vessels (Figure 4d). This implies that across neotropical tree taxa, xylem embolism resistance is related to the ability of the xylem vessels to resist hoop and bending stresses experienced during water transport. Recent evidence suggests that carbon starvation, induced by defoliation, can cause the development of fibers with thin walls which in turn results in irregular shaped vessels that are less resistant against embolism (Hillabrand et al., 2019). These results suggest that fibers with thick fiber walls can assist the bordering vessels to resist partial implosion, prevent stretching or rupture of the pit membranes and microfractures in the vessel walls, lowering the risk of embolism (Hillabrand et al., 2019; Jacobsen et al., 2005). The exact causal relationships between wood fibers, vessel bending resistance and xylem embolism resistance are not entirely clear but seem crucial in understanding embolism sensitivity in neotropical plant communities.

### 4.3 What are the drivers of drought‐induced mortality?

We find that the hydraulic safety margin is strongly related to the wood density specific hydraulic safety (the ratio between *P*_50_ and wood density) providing new insights into the interaction between wood density and embolism resistance in determining drought resistance (Figure 5). Species specific drought‐induced mortality was best explained by the hydraulic safety margin, confirming earlier findings from global meta‐analyses (Adams et al., 2017; Anderegg et al., 2016). Increasing wood density results in a decline of sapwood capacitance and therefore minimum branch xylem water potential. As a result, high wood density trees show a stronger desiccation of the leaves and stem during drought (Borchert, 1994; De Guzman et al., 2017; Meinzer, Woodruff, et al., 2008; Sterck et al., 2014). For this reason, embolism resistance has to increase with increasing wood density to maintain a wide enough hydraulic safety margin (Figure 5). However, many late‐successional evergreen species of wet tropical forests (e.g. *Eschweilera coriacea*, *Eschweilera sagotiana*, *Goupia glabra*, and *Eperua falcata*) did not show an increase of embolism resistance with wood density as a large proportion of the wood mass is allocated to axial parenchyma (Figure 3a; Figure S4). These species showed a low wood density specific hydraulic safety and high vulnerability to drought‐induced mortality (Figure 6). Moreover, many of these high wood density embolism sensitive tree species are considered hyperdominant species in the Amazon Basin (Fauset et al., 2015). The high wood density embolism sensitive group of tree species in this study represents four times as much aboveground biomass in the RAINFOR plots covering the Amazon Basin as the high wood density embolism resistant group (Figure S5).

### 4.4 What are the advantages of xylem volume allocation to axial parenchyma?

Embolism sensitive tree species generally experience a lower baseline mortality (Figure 6d) and show a preferential allocation of wood volume to axial parenchyma at the expense of fiber walls (Figure 6a). Therefore, we hypothesize that abundant axial parenchyma might promote tree longevity when seasonal drought is not a strong selection factor, as can be expected in ever wet tropical forests. Besides potential hydraulic benefits of axial parenchyma discussed previously, a high xylem volume fraction allocated to axial parenchyma might also be related to the storage of non‐structural carbohydrates (NSCs). In temperate tree species, the ray and axial parenchyma volume fraction of the wood is directly proportional to the storage of NSCs (Plavcová, Hoch, Morris, Ghiasi, & Jansen, 2016). Furthermore, among tropical tree species in Bolivia, the storage of NSCs is on average lower in species of dry environments compared to species of wet environments, that are expected to use these NSCs to survive in the deeply shaded understory (Poorter & Kitajima, 2007). Additionally, the high axial parenchyma fraction in shade‐tolerant understory trees might aid a rapid recovery through effective wound closure (Romero & Bolker, 2008) and vegetative resprouting (Pratt et al., 2007) following mechanical damage induced by falling debris or wind (Van Gelder et al., 2006). Finally, a high axial parenchyma fraction might prevent carbon starvation through the homeostatic maintenance of NSCs, as has been recently observed in tropical wet forest trees (Dickman et al., 2018).

If the high axial parenchyma fraction observed in many wet tropical tree taxa is indeed related to hydraulic efficiency and the storage and homeostatic maintenance of NSCs, we can assume that prioritizing axial parenchyma over fiber walls is an appropriate adaptation in ever wet and shaded tropical forests. However, while this evolutionary strategy might have been beneficial under past climatic conditions, it can become unsuited in a warmer and dryer climate. Enhanced drought‐induced mortality of high wood density embolism sensitive tree taxa might be driving observed compositional changes in neotropical forests (Esquivel‐Muelbert et al., 2018). Furthermore, increased drought‐induced mortality might underlie the observed trend of increased tree mortality observed in the Amazon Basin (McDowell et al., 2018) and the associated decline of the Amazon carbon sink strength (Brienen et al., 2015). Given the high drought sensitivity of many neotropical tree species of wet forests, future warming and drying could lead to the sudden collapse of standing biomass and the reversal of the carbon sink function of neotropical forests into a carbon source, enhancing global climate warming.

## Supporting information


**Appendix** **S1**: Supporting Information

**Appendix** **S2**: Supporting Information

**Appendix** **S3**: Supporting Information
