# Supplementary material for: Wood allocation trade‐offs between fiber wall, fiber lumen, and axial parenchyma drive drought resistance in neotropical trees
Source: Plant Cell Environ. 2020 Feb 3;43(4):965–80. doi: 10.1111/pce.13687 (PMC7155043; doi:10.1111/pce.13687)
Supplement: Supplementary file 1 — Appendix S1: Supporting Information [file PCE-43-965-s001.docx]

## Supporting Information

Article title: Wood allocation trade-offs between fiber wall, fiber lumen and axial parenchyma drive drought resistance in neo-tropical trees

Authors: Thomas Janssen, Teemu Hölttä, Katrin Fleischer, Kim Naudts and Han Dolman

The following Supporting Information is available for this article:

**Fig. S1** Relationship between xylem water potential at 50% loss of hydraulic conductivity (P_50_) and native state embolism.

**Fig. S2** Relationship between minimum leaf and branch xylem water potential.

**Fig. S3** Relationship between species averaged length of longest continuous vessel and the xylem volume allocated to axial parenchyma.

**Fig. S4** Relationship between wood axial parenchyma density and xylem water potential at 50% loss of conductivity (P_50_).

**Fig. S5** Relationships between wood fiber density and measures of plant performance.

**Fig. S6** Xylem volume allocation strategies and importance of taxa across the Amazon.

**Table S1** Database overview.

**Fig. S1** Relationship between xylem water potential at 50% loss of hydraulic conductivity (P_50_) and native state embolism. **
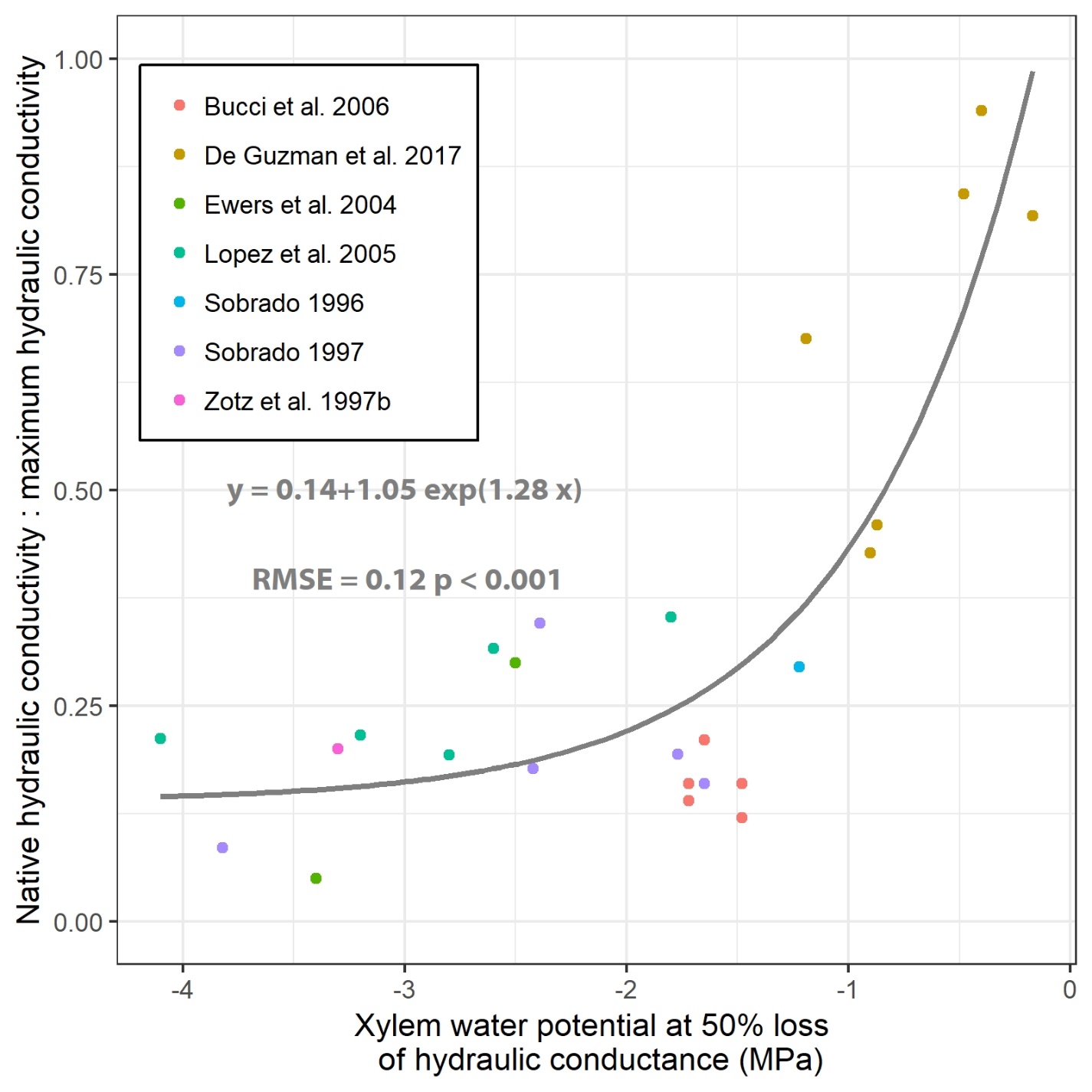
**

**Fig. S2** Relationship between minimum leaf and branch xylem water potential. The black dashed line indicates the 1:1 line and shows that leaf water potential is always lower compared to branch water potential.
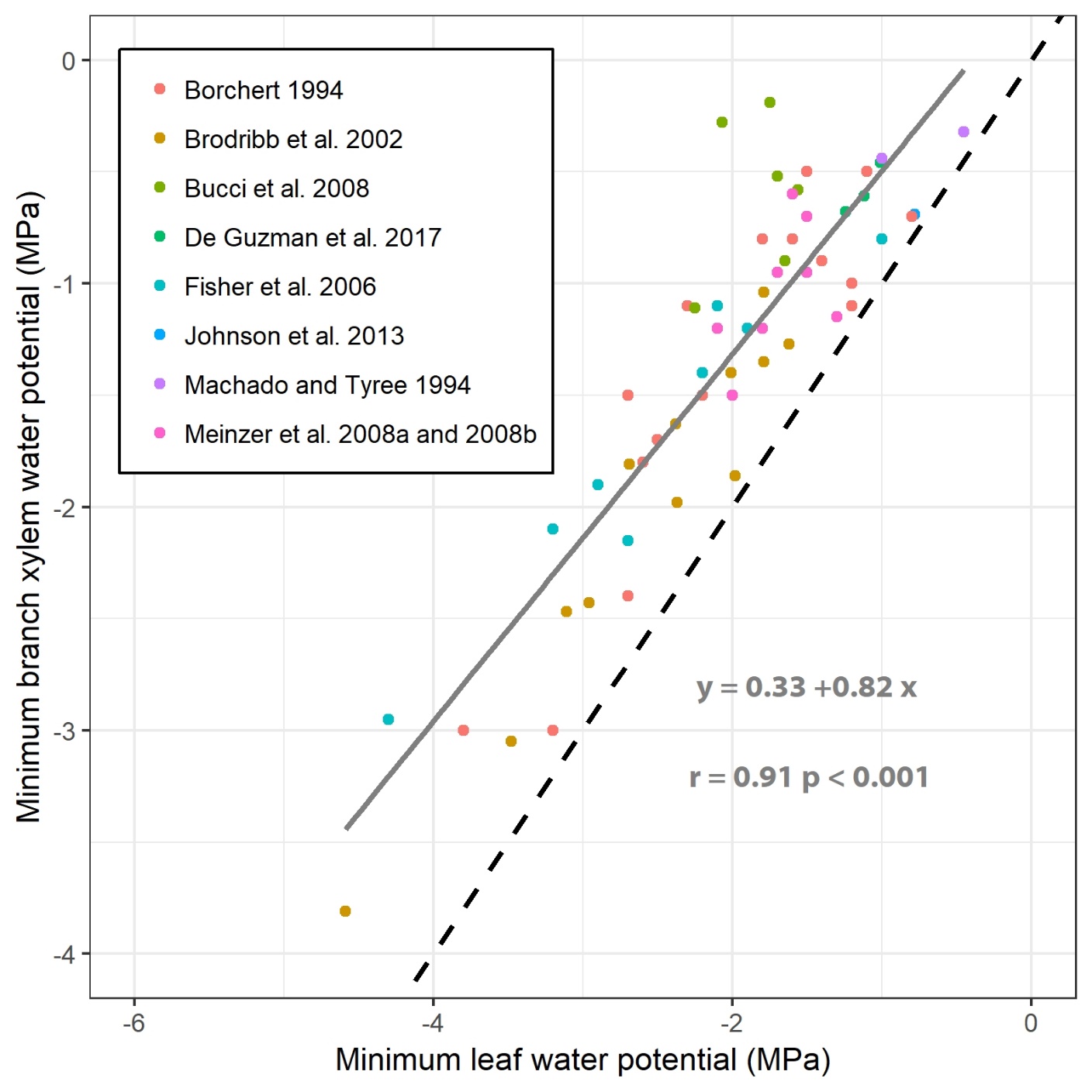


**Fig. S3** Relationship between species averaged length of longest continuous vessel and the xylem volume allocated to axial parenchyma.
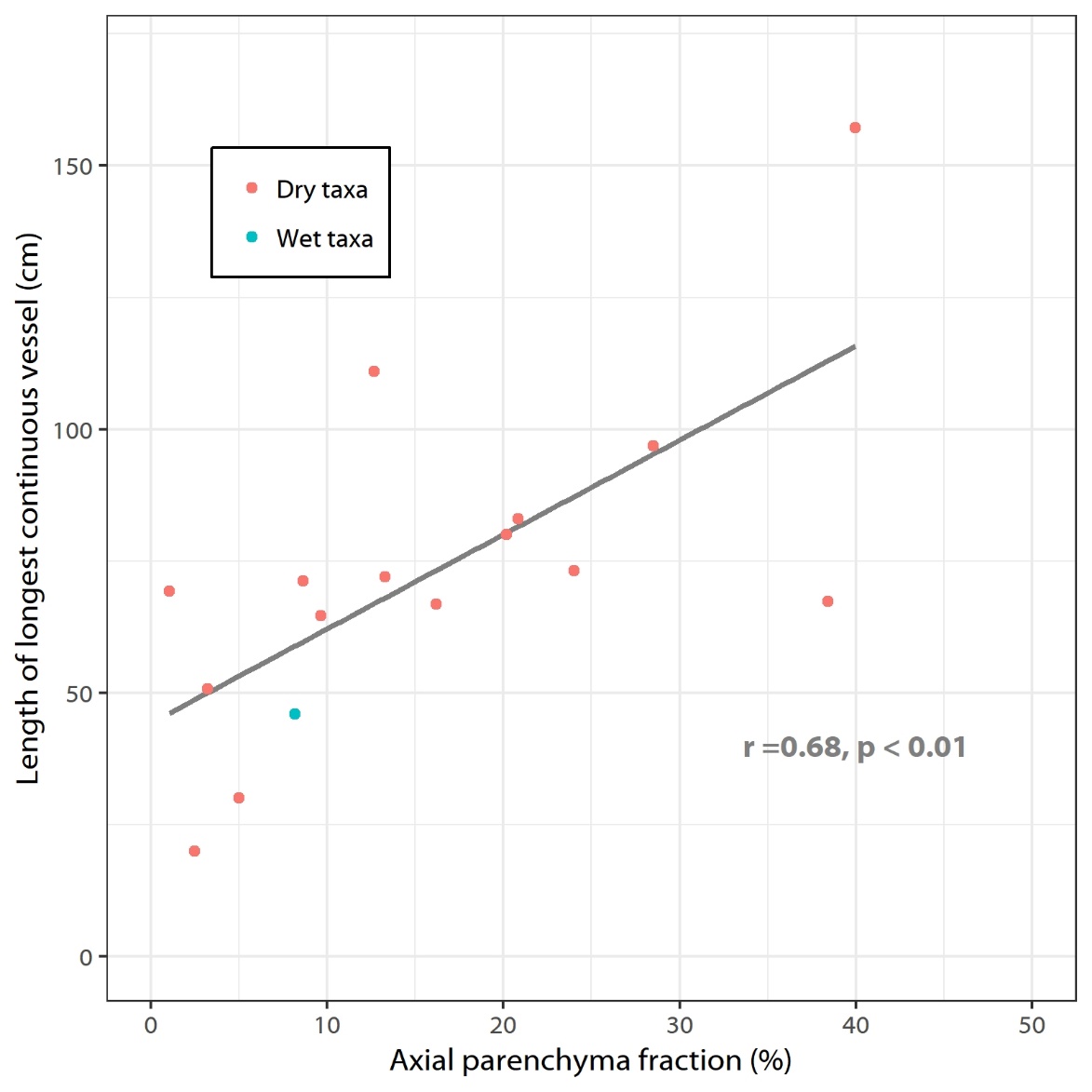


**Fig. S4** Relationship between wood axial parenchyma density and xylem water potential at 50% loss of conductivity (P_50_).
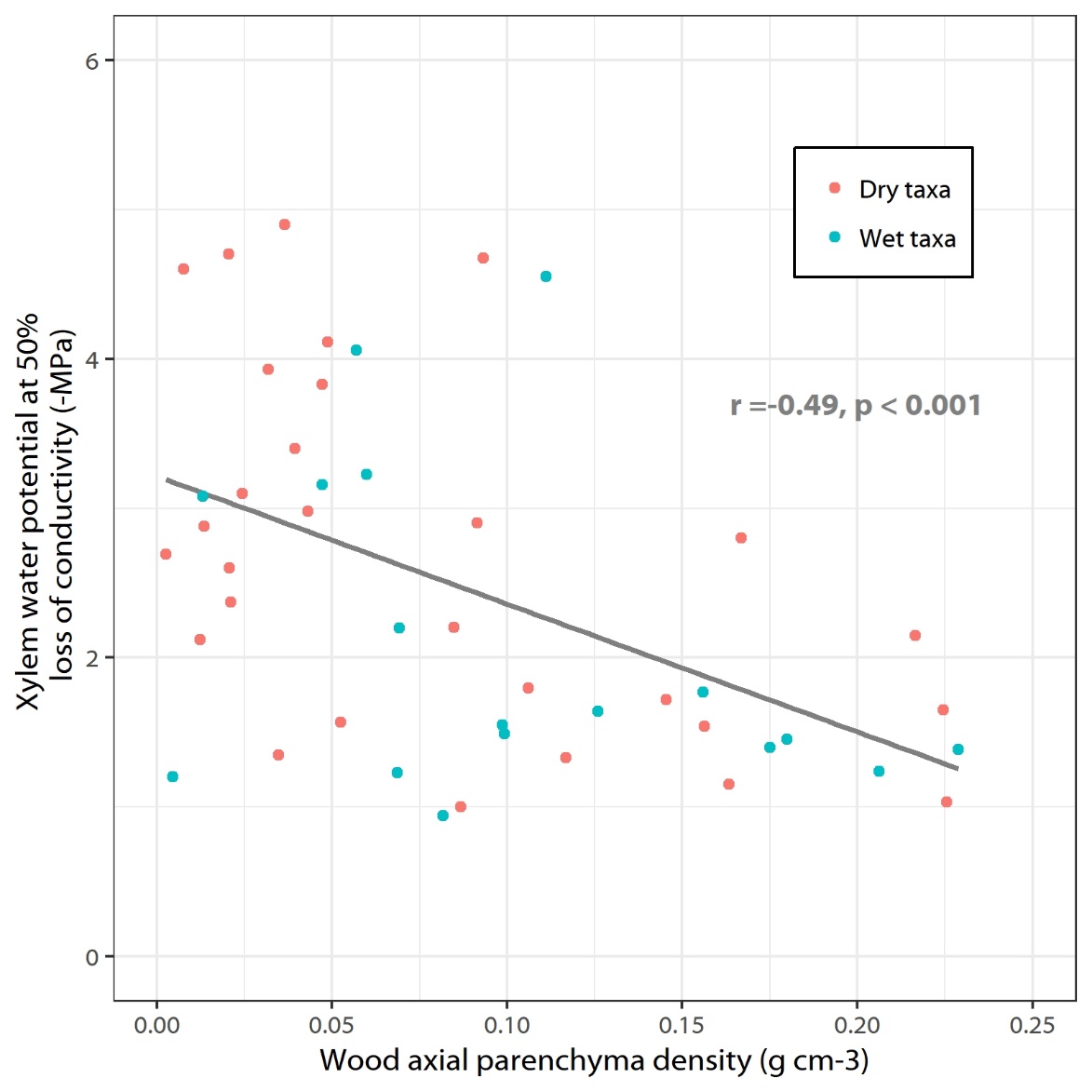


**Fig. S5** Relationships between wood fiber density and measures of plant performance.
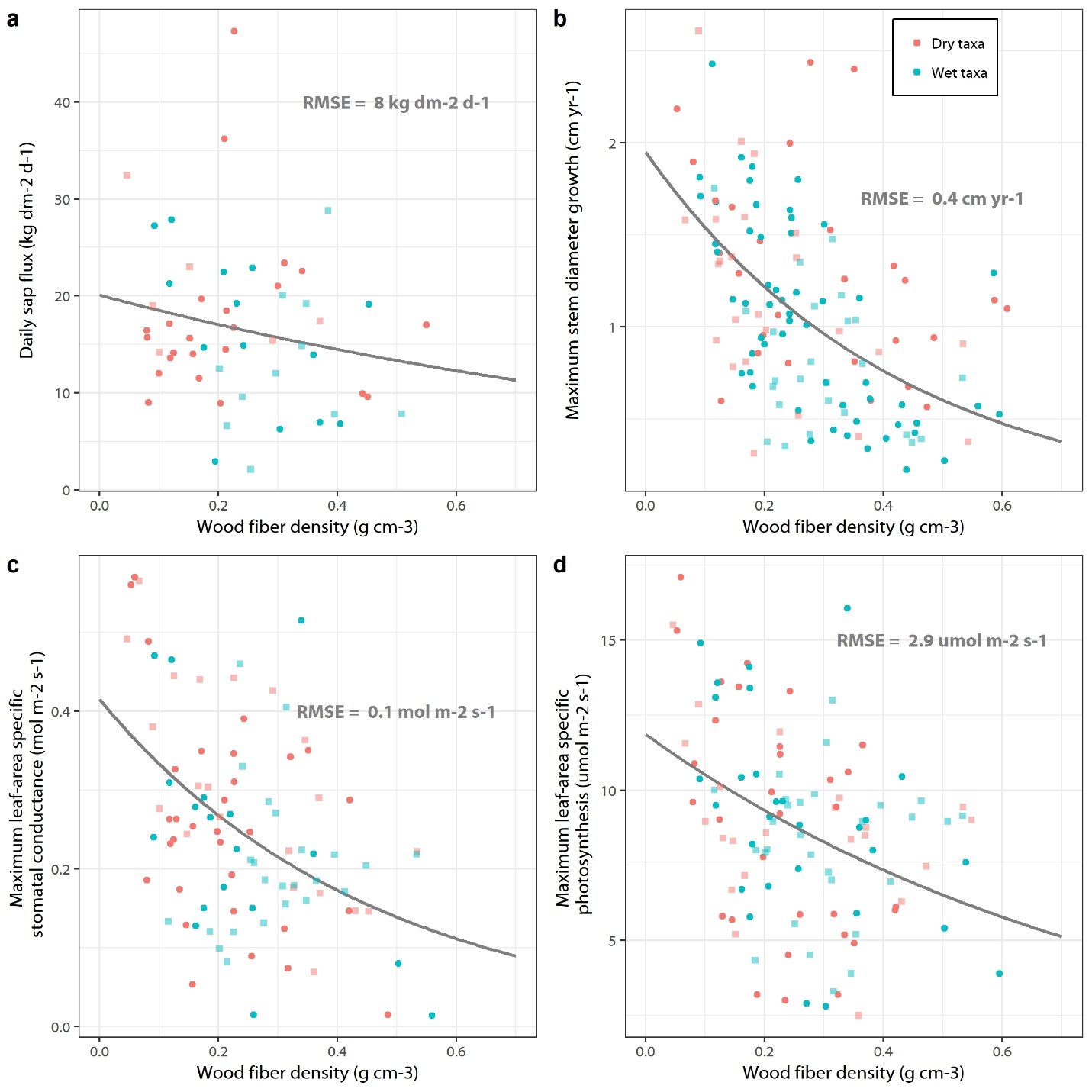


**Fig. S6** Xylem volume allocation strategies and importance of taxa across the Amazon. The xylem tissue fractions are: fiber lumen (Fl) and wall (Fw), axial parenchyma (Pa) and ray parenchyma (Pr), vessel wall (Vw) and vessel lumen (Vl).
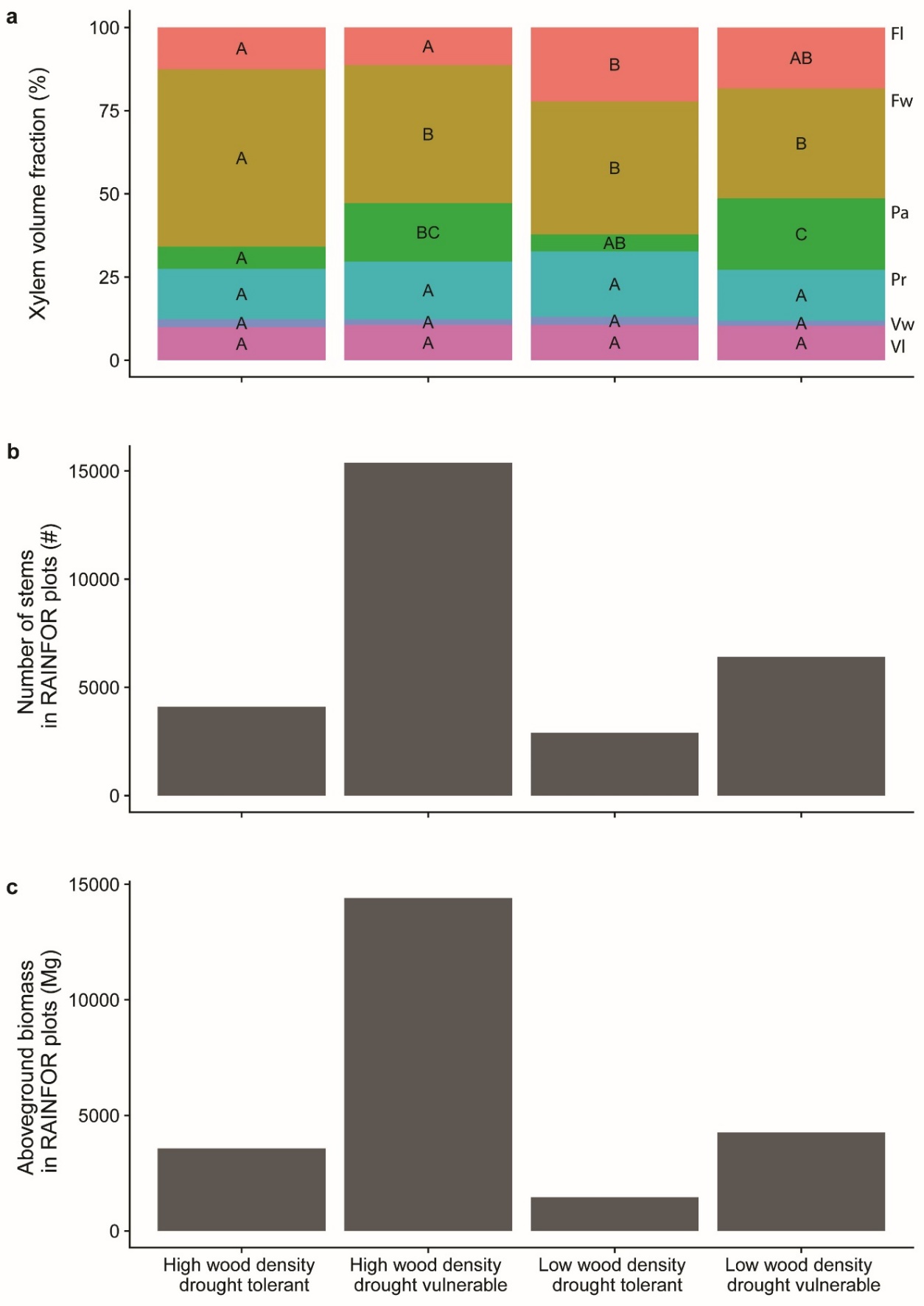
.

**Table S1** Database overview. Number of unique species, genera, individuals, sites and sources for the different xylem traits, xylem (hydraulic) traits and measures of plant performance and hydraulic status included in the database. * denote the number of observations that were added by estimating the particular variable

|  | **Measure** | **Units** | **Species** | **Genus** | **Individuals** | **Sites** | **Sources** |
| --- | --- | --- | --- | --- | --- | --- | --- |
| **Xylem traits** | Vessel lumen diameter | µm | 1177 | 469 | 2210 | 75 | 132 |
|  | Vessel density | # mm^-2^ | 1162 | 468 | 2005 | 72 | 127 |
|  | Vessel wall thickness | µm | 206 | 108 | 228 | 17 | 34 |
|  | Mean longest vessel | cm | 121 | 102 | 126 | 9 | 15 |
|  | Fiber lumen diameter | µm | 471 | 250 | 620 | 28 | 77 |
|  | Fiber wall thickness | µm | 601 | 297 | 841 | 34 | 84 |
|  | Fiber wall fraction | % | 246 | 173 | 261 | 12 | 41 |
|  | Fiber lumen fraction | % | 273 | 179 | 300 | 13 | 44 |
|  | Fiber fraction | % | 350 | 198 | 475 | 21 | 59 |
|  |  |  | 135* | 105* | 162* | 2* | 11* |
|  | Ray parenchyma fraction | % | 264 | 170 | 328 | 14 | 54 |
|  | Axial parenchyma fraction | % | 260 | 165 | 324 | 14 | 55 |
|  | Parenchyma fraction | % | 418 | 241 | 549 | 19 | 59 |
|  |  |  | 69* | 59* | 89* | 6* | 11* |
|  | Vessel wall fraction | % | 227 | 118 | 252 | 17 | 37 |
|  | Vessel lumen fraction | % | 227 | 118 | 252 | 17 | 37 |
|  | Vessel fraction | % | 453 | 261 | 859 | 33 | 93 |
| **Xylem (hydraulic) properties** | Wood density | g cm^-3^ | 2280 | 644 | 4027 | 74 | 118 |
|  | Xylem water potential at 50% loss of hydraulic conductance (P_50_) | MPa | 264 | 163 | 437 | 29 | 52 |
|  | Measured maximum sapwood area specific hydraulic conductivity | kg m^-1^ s^-1^ MPa^-1^ | 168 | 126 | 278 | 19 | 29 |
|  |  |  | 138* | 103* | 292* | 13* | 17* |
|  | Sapwood specific hydraulic capacitance | kg m^-3^ MPa^-1^ | 113 | 85 | 146 | 10 | 16 |
| **Plant hydraulic status and plant performance** | Minimum xylem water potential of terminal branches | MPa | 62 | 54 | 108 | 8 | 13 |
|  |  |  | 329* | 198* | 614* | 30* | 64* |
|  | Maximum leaf area specific stomatal conductance | mol m^-2^ s^-1^ | 255 | 168 | 508 | 20 | 47 |
|  | Maximum leaf area specific photosynthesis | µmol m^-2^ s^-1^ | 443 | 238 | 606 | 35 | 32 |
|  | Maximum stem diameter growth | cm year^-1^ | 850 | 302 | - | - | 1 |
|  | Sapwood area specific daily transpiration in the wet season | Kg dm^-2^ d^-1^ | 91 | 73 | 161 | 13 | 25 |
|  | Drought-induced mortality index | Index | 350 | 194 | 587 | 8 | 8 |
